# Nonclassical yellow nail syndrome in six-year-old girl: a case report

**DOI:** 10.1186/1757-1626-2-165

**Published:** 2009-10-24

**Authors:** Filiz Cebeci, Muge Celebi, Nahide Onsun

**Affiliations:** 1 Department of Dermatology, Vakif Gureba Training and Research Hospital, İstanbul, Turkey

## Abstract

**Introduction:**

The yellow nail syndrome is usually described as the combination of yellow nails with lymphoedema and often with respiratory manifestations such as pleural effusions, chronic sinusitis and bronchiectasis. The syndrome is most often seen in the middle-aged individuals.

**Case presentation:**

We present a 6-year-old girl with yellow nail syndrome having pansinusitis and bronchiectasis.

**Conclusion:**

The components comprising the classical triad of yellow nail syndrome in children may not necessarily be present altogether. Therefore, yellow nail syndrome should be suspected in children having only typical nail changes.

## Introduction

Yellow nail syndrome (YNS) is a rare clinical entity consisting of dystrophic yellow nails, lymphoedema and pleural effusion [1,2]. In addition, a significant number of patients also have sinusitis [3], bronchiectasis [4], and lower respiratory tract infections [5]. It has been reported that congenital malformations and a secondary dysfunction of lymphatic vessels may be responsible for the syndrome including changes of nail, but its exact mechanism is still not known [6]. The syndrome is most often seen in middle-aged individuals. Here we present a six-year-old girl aside from the classic triad of the syndrome having pansinusitis and bronchiectasis.

## Case presentation

A 6-year-old girl presented with thickening and slow-growing nails of both hands and feet, and change in color of nails which had been noted since her birth according to history given by her parents. In her history, there was no known history about sinusitis and bronchiectasis apart from that of recurrent bronchopneumonia attacks. Physical examination revealed lymphadenopathy which was bilateral, submandibular, mobile, and 0.5 cm in size. While expirium was prolonged; bilateral, partial, thin ralles and rhoncus were heard over the lung on inspirium.

**Figure 1:**
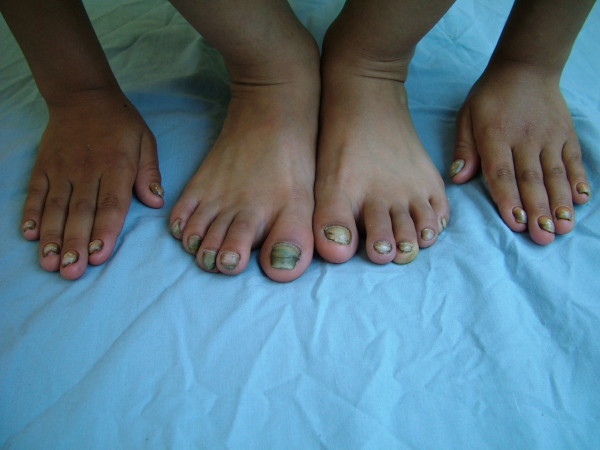
**Yellow-greenish discoloration, thickening of the nail plate, and disappearance of the cuticle on both hands and feet in the six-year-old-girl**.

**Figure 2:**
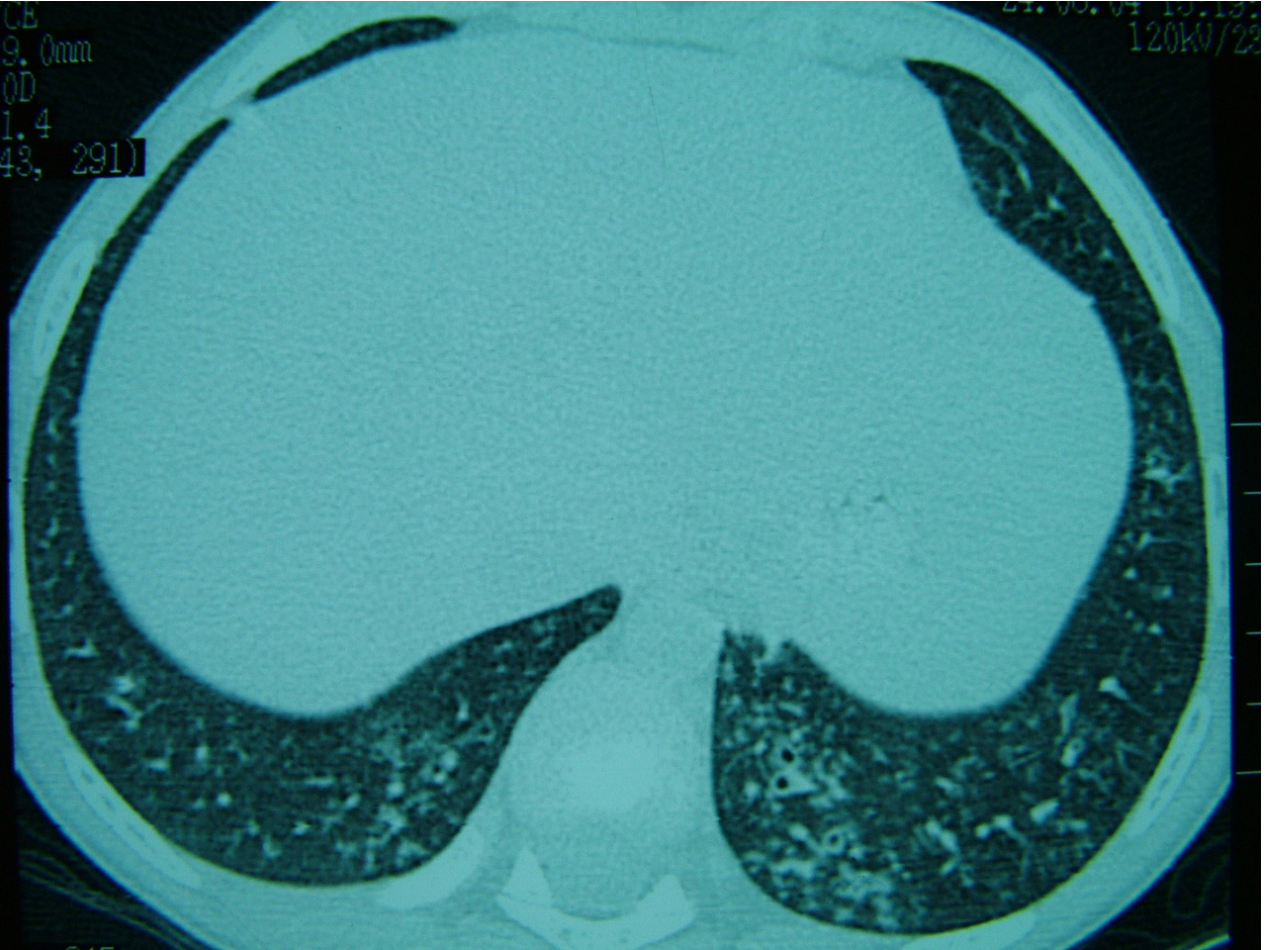
**High resolution computerized tomography revealing bronchiectasis**.

**Figure 3:**
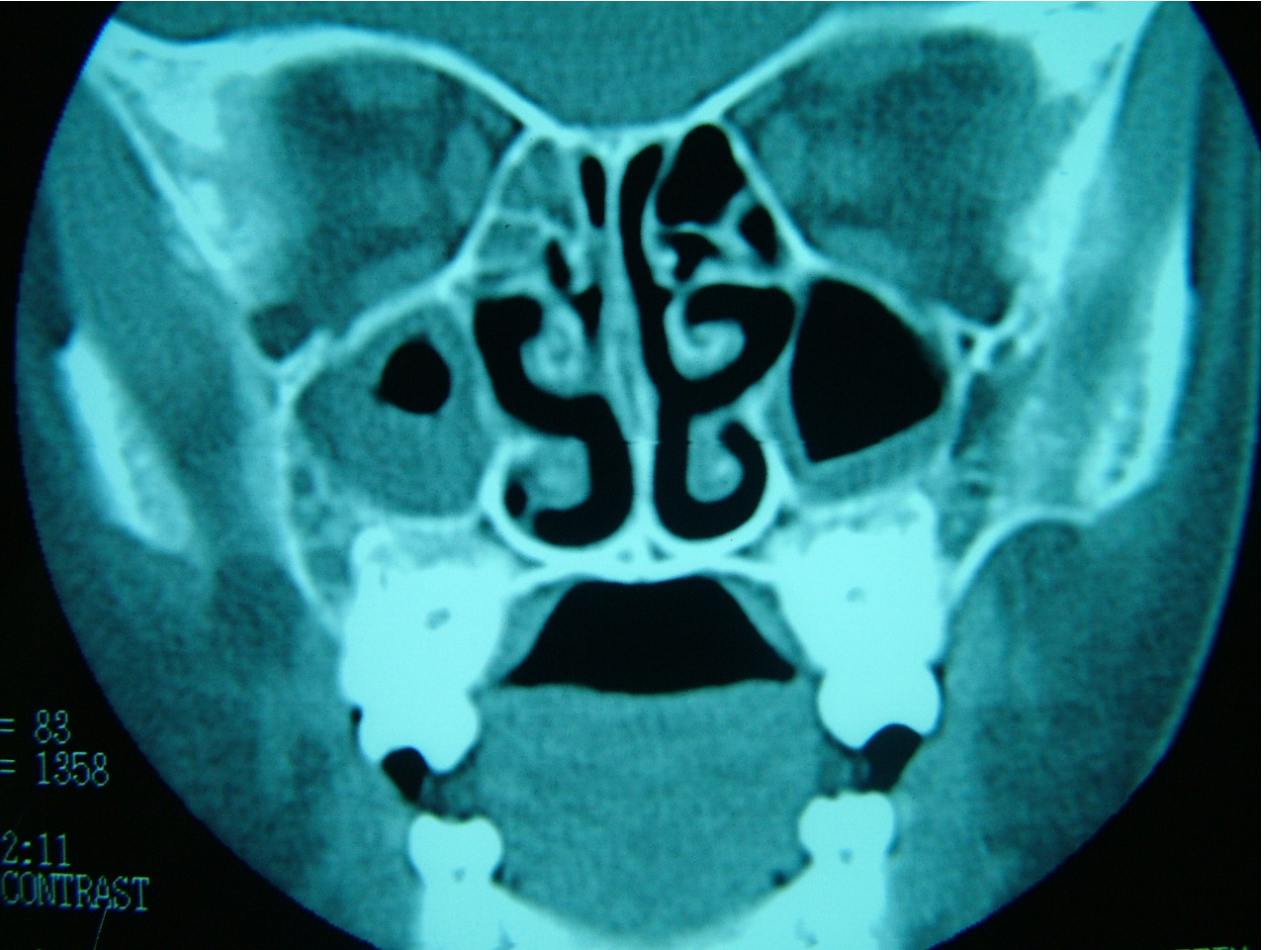
**Paranasal computerized tomography revealing pansinusitis**.

On dermatologic examination, all of her nails showed a yellow-greenish discoloration with thickening of the nail plates, excessively curving a long both axes and loss of the cuticles (Fig. 1). Laboratory studies were normal except for erythrocyte sedimentation rate (40 mm/hour), including complete blood count, CRP, HIV testing, thyroid functional tests, protein electrophoresis, tumor markers, routine biochemistry and urinalysis. Direct microscopic examination and culture of the nail clippings were negative for fungi. The patient was consulted by pulmonologist. Chest radiograph revealed some patchy non-homogeneous densities in the right paracardiac and perihilar areas and left paracardiac areas. High resolution computerized tomography demonstrated tubular bronchiectatic areas in patches filled with mucus pluggings in the posterobasal segments of the left and right lower lobes and aciner-nodular style infiltrations in the adjacent lung parenchyma (Fig. 2). That pulmonary function tests performed were found to be normal was assessed that the obstruction was not developed yet. Bilateral submandibular lymphadenopathy, deviation of septum nasy to left and postnasal secretion were present on the otorhinolaryngology examination. On the paranasal computerized tomography, the frontal sinus was found out to be in the developmental period. In the right and left anterior etmoid sinuses, more evident on the right side, the existence of widespread mucosal thickenings and lack of ventilation at both maxillary sinuses and sphenoid sinus had been evaluated as pansinusitis (Fig. 3). Lymphoedema was not observed in our case and lymphocintigraphy performed was normal. Furthermore, the patient's abdominal ultrasonography was normal.

## Conclusion

The syndrome was initially described in 1964, in a study of thirteen patients with yellow nails and lymphedema [1]. Two years later, Emerson added pleural effusion as a frequent feature of the syndrome. The whole syndrome consist of the classical triad including yellow nails, lymphoedema and pleural effusions with associated respiratory tract involvement [2]. Furthermore, bronchiectasis [3], and rhinosinusitis [4] are well recognized respiratory manifestations of this syndrome. YNS may be associated with a number of systemic diseases such as rheumatoid arthritis, thyroiditis, acquired immunodeficiency syndrome, tuberculosis, immunologic disorders, malignancies and mycosis fungoides [7]. YNS is a rare disease and its exact pathogenesis is still not known. The disease is believed to start with congenital lymphatic blood vessel hypoplasia and inadequate lymphatic drainage system [6] and it is responsible for all the findings including nails. The disease is also considered to have a genetic component, although it has not been proved yet [5]. Although there are not similar findings on the other people in the family of our case, the nail findings were expressed to be present from the birth of the child.

The mean age at onset is 40 years. However, the age of onset varies widely; for example lymphoedema may be present even from the birth until 60's and it rarely affects children. The yellow nails are found in 89%, lympoedema in 80% and pleural effusion in 36% of the cases. These three findings are concurrently seen totally only in one, third of patients [8]. In 1972 it was stated that existance two finding are enough to establish the diagnosis [9]. Moreover, only typical nail changes are enough for the diagnosis of YNS [10]. In most cases, all of 20 nails are affected. The nails are typically yellowish green in color, very slow growing, thickened and excessively curved a long both axes. The cuticle and lanula are usually absent and onycholysis is frequently evident [1,7,8]. Our case was a 6-year-old girl and and findings of the nails was said to have been present from the birth onwards.

In 1969 Bower et al. suggested the bronchiectasis as the fourth compenenet. Bronchiectasis is considered as possibly related with the hypoplasia of the bronchial lymphatics in YNS. Pre-exisiting lymphatic anomaly possibly tends to cause recurrent respratory infection and bronchiectasis; and damage caused by recurrent inflamation later predisposes some patients to develop pleural effusion. There was history of having former respiratory infection and we established bronchiectasis. Here, it appeared that bronchiectasis was probably the early manifestation of this syndrome. Since our case is already six years old pleural effusion and lymphoedema are expected to occur later. In 1994 rhinosinusitis may be recognised as part of the syndrome and rhynosinusitis frequency has been reported 83% [4]. She had similar nail findings, pansinusitis and bronchiectasy but had not pleural effusion, lymphedema.

No specific treatment exists for this disorder. The patient was given 300 IU/day vitamin E, for three months but the patient did not respond to treatment. We presented a case with the exception of classical triad.

## Consent

Written informed consent was obtained from the patient's mother for publication of this case report and accompanying images. A copy of the written consent is available for review by the Editor-in-Chief of this journal.

## Competing interests

The authors declare that they have no competing interests.

## Authors' contributions

FC conceived of the case report, and participated in its design and coordination. MC performed management and consultation of the case. NO participated in the sequence alignment and drafted the manuscript.

## References

[B1] Samman PD White WF The "yellow nail syndrome" Br J Dermatol 1964 76 153 157 10.1111/j.1365-2133.1964.tb14499.x 14140738

[B2] Emerson PA Yellow nails, lymphoedema, and pleural effosions Thorax 1966 21 247 253 10.1136/thx.21.3.247 5914998 PMC1019033

[B3] Bowers D Unequal breasts, yellow nail, bronchiectasis and lymphedema Can Med Assoc J 1969 100 437 438 5767837 PMC1945734

[B4] Varney VA Cumberworth V Sudderick R Durham SR Mackay IS Rhinitis, sinusitis and the yellow nail syndrome: a review of symptoms and response to treatment in 17 patients Clin otolaryngol 1994 19 237 240 10.1111/j.1365-2273.1994.tb01222.x 7923847

[B5] Battaglia A Di Ricco G Mariani G Giuntini C Pleural effosion and recurrent broncho-pneumonia with lymphedema, yellow nails and protein-losing enteropathy Eur J Respir Dis 1985 66 65 9 3979479

[B6] Bull RH Fenton DA Mortimer PS Lymphatic function in the yellow nail syndrome Br J Dermatol 1996 134 307 312 10.1111/j.1365-2133.1996.tb07619.x 8746347

[B7] Hershko A Hirshberg B Nahir M Friedman G Yellow nail syndrome Postgrad Med J 1997 73 466 468 10.1136/pgmj.73.862.466 9307736 PMC2431365

[B8] Nordkild P Kromann-Andersen H Struve-Christensen E Yellow nail syndrome. The triad of yellow nails, lymphedema and peural effusions: a review of the literature and a case report Acta Med Scand 1986 219 221 227 3962735 10.1111/j.0954-6820.1986.tb03302.x

[B9] Hiller E Rosenow EC III Olsen AM Pulmonary manifestations of the yellow nail syndrome Chest 1972 61 452 458 10.1378/chest.61.5.452 5046843

[B10] Bolognia JL Jorizzo JL Rapini RP Dermatology 2008 2 Elsevier-Mosby, Spain18694683

